# Tanshinone IIA Protects Against Cerebral Ischemia Reperfusion Injury by Regulating Microglial Activation and Polarization *via* NF-κB Pathway

**DOI:** 10.3389/fphar.2021.641848

**Published:** 2021-04-19

**Authors:** Zhibing Song, Jingjing Feng, Qian Zhang, Shanshan Deng, Dahai Yu, Yuefan Zhang, Tiejun Li

**Affiliations:** ^1^ Department of Pharmacy, Punan Hospital, Pudong New District, Shanghai, China; ^2^ College of Pharmacology, Anhui University of Chinese Medicine, Hefei, China; ^3^ School of Medicine, Shanghai University, Shanghai, China; ^4^ 970 Hospital, Weihai, China

**Keywords:** tanshinone IIA, cerebral ischemia, microglia, polarization, inflammation

## Abstract

Tanshinone IIA, a fat-soluble diterpenoid isolated from Salvia miltiorrhiza Bunge, has been shown to attenuate the cerebral ischemic injury. The aim of this study was to examine the effects on neuroprotection and microglia activation of Tanshinone IIA. Male Sprague-Dawley rats were subjected to middle cerebral artery occlusion (MCAO). We found that Tanshinone IIA significantly reduced infarction volume, alleviated neuronal injuries, reduced the release of TNF-α, IL-1β, and IL-6, increased SOD activity, and decrease the content of MDA in MCAO rats. Hematoxylin and eosin staining, Nissl staining, TUNEL staining and immunofluorescence staining showed that Tanshinone IIA improved the distribution and morphology of neurons in brain tissues and reduced apoptosis. In addition, Co-immunofluorescence staining of rat brain tissues and the mRNA expression levels of CD11b, CD32, iNOS, and Arg-1, CD206, IL-10 in BV2 cells indicated that Tanshinone IIA can downregulate M1 microglia and upregulate M2 microglia in MCAO rats. Further, BV2 microglial cells were subjected to oxygen-glucose deprivation, the protein expression levels were detected by western blot. Tanshinone IIA inhibited the expression levels of NF-κB signaling pathway related proteins. Taken together, this study suggested that Tanshinone IIA modulated microglial M1/M2 polarization *via* the NF-κB signaling pathway to confer anti-neuroinflammatory effects.

## Introduction

Senile neurological diseases stroke has become the third leading cause of death in developed countries and the main cause of global disability (Collaborators, 2018). Among stroke patients, stroke caused by cerebral artery thrombosis or cerebral ischemia accounts for 80–85% (Alves et al., 2019). The molecular mechanism of stroke pathology is very complex as this process includes oxidative stress, blood-brain barrier destruction, apoptosis, and inflammatory response. It causes brain swelling and leads to brain cell death (Wahul et al., 2018).

The main pathophysiological mechanism of ischemic stroke neurological injury is inflammatory response. Microglia are one of the resident immune cells in the central nervous system. Affected by its environment, microglia have multiple phenotypes and retain metastatic function to maintain tissue homeostasis (Orihuela et al., 2016). Similar to macrophages, microglia’s polarization can be divided into different phenotypes; M1 and M2 type (Hansen et al., 2018). Traditionally, in the case of ischemic stroke, microglia rapidly transform from a resting state to an active “amebic-like” state, releasing a variety of inflammatory and cytotoxic mediators, causing cell damage and death, thus aggravating brain damage (Zhao et al., 2017; Lamkanfi and Dixit, 2014). M2 type can promote nerve repair during central nervous system injury (Chen et al., 2015; Kalkman and Feuerbach, 2016; Liu et al., 2019) and has a vital role in resolving inflammatory responses and debris removal, tissue remodeling, and angiogenesis (Mantovani et al., 2013).

The traditional Chinese medicine Radix Salviae Miltiorrhizae belongs to the blood-activating and stasis-relieving medicine. Tanshinone IIA, a fat-soluble diterpenoid isolated from Salvia miltiorrhiza Bunge, is one of the main active components of this red sage. It has several pharmacological effects, such as anti-atherosclerosis and myocardial protection, anti-platelet aggregation, and anti-tumor effect (Fei et al., 2017; Pan et al., 2017; Ye et al., 2020). Several studies have shown that Tanshinone IIA has neuroprotective effects against cerebral ischemia injury (Wang et al., 2014.; Chen et al., 2019). Over the past decade, Tanshinone IIA was demonstrated to have potential protective effects against cerebral ischemia-reperfusion injury through a variety of mechanisms (Zhu et al., 2017; Ma et al., 2019; He et al., 2020; Huang et al., 2020). Tanshinone IIA decreased the expression of cleaved caspase-3 protein and increased the expression of B-cell lymphoma 2 (bcl-2) protein in the ischemic cortex (Chen et al., 2012b). This would indicate that the neuroprotective effects of Tanshinone IIA against focal cerebral ischemic/reperfusion injury are likely to be related to the attenuation of apoptosis. Another study showed that Tanshinone IIA exerts neuroprotective effects through activation of nuclear factor erythroid 2-related factor-dependent antioxidant responses (Cai et al., 2017). A recent study reported that Tanshinone IIA can reduces the number of inflammatory cells in the brain after cerebral ischemia-reperfusion and decreases ischemia-induced upregulation of autophagy-related proteins, such as such as LC3-II, Beclin-1 and Sirt 6 (Wang et al., 2020). These findings suggest that Tanshinone IIA can provide protection against ischemic stroke by inhibiting inflammation and autophagy. However, there is no report on its effect on microglia after cerebral ischemia-reperfusion injury, and the mechanism of its neuroprotection is unclear.

The main objective of this study was to investigate the neuroprotective effect of Tanshinone IIA on rat brain after ischemia-reperfusion injury and its effect on microglia, and to further demonstrate the potential mechanism.

## Materials and Methods

### Reagent

Tanshinone IIA (T4952) and 2,3,5-triphenyltetrazolium chloride (TTC) were purchased from Sigma-Aldrich (United States).; BPL (3-n-Butylphthalide, a marketed drug for acute ischemic stroke) (H20100041) was purchased from CSPC (China); BAY-11-7082 (a NF-κB inhibitor) (S2913) was purchased from Selleck (United States); ELISA Kit was purchased from R&D Systems (United States); superoxide dismutase (SOD) and malondialdehyde (MDA) assay kit were purchased from Nanjing Jiancheng Bioengineering Institute (China); total RNA Kit was purchased from Takara (Japan). Primary antibodies: NeuN (24307), Iba-1 (17198), iκB (76041), NF-κB (p65) (8242), GAPDH (5174) were purchased from Cell Signaling Technology (United States); p-iκB (ab133462) and p-NF-κB (p-p65) (ab239882) were purchased from Abcam (United States); CD16/32 (AF1460) and CD206 (AF2535) were purchased from R&D Systems (United States). Secondary antibodies: Alexa Fluor^®^ 488 AffiniPure Goat Anti-Rabbit IgG, Alexa Fluor^®^ 488 AffiniPure Donkey Anti-Goat IgG and Cy™3 AffiniPure Donkey Anti-Rabbit IgG were purchased from Jackson (United States).

### Animal Care and Experimental Protocol

Male Sprague-Dawley (SD) adult rats (∼250 g) were purchased from Slack Laboratory Animals Co., Ltd. (Shanghai, China). Food and water were available under constant temperature (23 ± 2°C) and controlled light conditions (12 h light/dark cycle). All experiments were in accordance with the guidelines of the Animal Care Committee of Shanghai University. During the experiment, all experimental animals were treated humanized.

All rats were divided randomly into six groups: Sham group (sham-operation treated with 0.9%NaCl, N = 12); MCAO group (MCAO treated with 0.9%NaCl, N = 12); BPL group (MCAO treated with 10 mg/kg butyphthalide, N = 12); TanIIA 1 mg/kg group (MCAO treated with 1 mg/kg Tanshinone IIA, N = 12); TanIIA 3 mg/kg group (MCAO treated with 3 mg/kg Tanshinone IIA, N = 12); and TanIIA 9 mg/kg group (MCAO treated with 9 mg/kg Tanshinone IIA, N = 12). After cerebral ischemia 10min, drugs were injected (tail vein injection). And after reperfusion, drugs were injected (tail vein injection) again.

### Middle Cerebral Artery Occlusion Model and Neurological Deficit Scores

Middle cerebral artery occlusion (MCAO) was performed according to the Longa’s method (Longa et al., 1989). SD rats were anesthetized with intraperitoneal 2% sodium pentobarbital (3 ml/kg). Then an incision was cut in the cervical midline. The exposed external carotid arteries were closed by sterile suture, and the vascular clamp was used to clamp internal carotid artery. Subsequently, an incision in the common carotid artery was made, the proximal common carotid artery is tightly tied, and the suture line (0.26°mm; Cinontech, China) is inserted into the skull along the internal carotid artery along the small hole, inserted into the black mark (∼18 mm), then the internal carotid artery is ligated. During surgery, the cerebral blood flow (CBF) in the left cortex of rats was monitored by Laser Doppler Flowmeter (Oxylab LDF; B&E TEKSYSTEMS LTD., China), and the reduction of CBF in rats to less than 20% of the initial value was a sign of successful surgery. After 2 h, the rats underwent reperfusion. Sham-operated rats receive identical surgery without arterial ligation and monofilament insertion. Animals were warmed under warm light before waking up.

After 24 h of reperfusion, the neurological deficit score was assessed. The neurological dysfunction in rats was assessed as previous (Longa et al., 1989) in a double-blinded manner with the following definitions: Score 0: no detectable neurological deficits; Score 1: failure to extend the right forepaw fully; Score 2: circling to the left; Score 3: falling to the left; Score 4: did not walk spontaneously and had a depressed level of consciousness.

### Assessment of Cerebral Infarct Volume

After analysis of neurological function, the rats were sacrificed by sodium pentobarbital injection and the brains were harvested. Brains were rinsed with saline to remove excess water, and immediately placed in the freezer for 15 min. Then forebrains were dissected into six sections in coronal plane (2-mm slice) (Chiang et al., 2011). The slices were placed in 2% TTC (Sigma- Aldrich, United States) for 20 min at room temperature. followed by fixation in 4% formaldehyde at 4°C for 24 h. Brain slices were photographed and analyzed by ImageJ analysis software (v6.0). The infarct volume was calculated as follows: the left infarct volume/whole brain volume × 100%.

### Hematoxylin and Eosin Staining

Rats were anesthetized, and perfused with physiological saline and 4% paraformaldehyde (Sinopharm, China) *via* cardiac puncture. Brains were harvested, fixed with 4% formaldehyde, embedded in paraffin, sliced into 5-μm-thick coronal sections. Slices were stained with hematoxylin for 3–8 min, washed with tap water, alcoholic fractionation with 1% hydrochloric acid for a few seconds, rinsed with tap water, returned to blue with 0.6% ammonia, and rinsed with running water, then slices were stained in eosin staining solution for 1–3 min. Areas of interest were observed with a light microscope.

### Nissl Staining

Paraffin sections went through xylene (Sinopharm, China) dewaxing and an alcohol gradient rehydration as above and stained with Nissl solution (Beyotime, China) for 5–10 min. Then slices were treated with 95% alcohol until Nissl bodies were dark blue and the background was light blue or colorless. They were placed under microscope for observing Nissl bodies in cortical neurons.

### TUNEL Staining

TUNEL staining was performed using an apoptosis kit (Roche, China) according to the manufacturer’s instructions. In brief, after the sections were dewaxed, proteinase K was added and incubated at 37°C for 30 min, the film-breaking solution was added dropwise and incubated at room temperature for 30 min, after washing, TUNEL reagent was added and incubated at 37°C for 1 h. Then the sections were incubated in 3% hydrogen peroxide solution at room temperature for 15 min, converter-POD was added and incubated at 37°C for 30 min, and finally DAB was added dropwise for color development. TUNEL-positive neurons surrounding the injury areas were observed and counted under high magnification on a fluorescence microscope (Olympus, Japan).

### Immunofluorescence Assessment

The sections were placed in a 0.1 M sodium citrate buffer solution (pH = 6.0) at 100°C for 30 min for antigen retrieval. After cooled to room temperature, the membrane was lysed for 5 min using 0.1% Triton X-100, sections were blocked with 1% bovine serum albumin (BSA, Sigma, MO, United States) + 0.1% TritonTM X-100 for 1 h and incubated at 4°C overnight with primary antibodies: NeuN (1:250) or Iba-1 (1:250), followed by incubation with appropriate fluorescence-conjugated secondary antibodies: Cy™ 3 AffiniPure Donkey Anti-Rabbit IgG (1:200) for 2 h in the dark. In addition, 4, 6-diamidino-2-phenylindole (DAPI) (Beyotime, China) was used to counterstain nuclei for 10 min in the dark, and then mounted with a sealing liquid. The ischemic lateral hemicortical region was observed by laser confocal microscopy and photographed. Fluorescence intensity was analyzed and calculated by ImageJ software (National Institutes of Health, Bethesda, MD, United States).

### Co-Immunofluorescence Staining

The brain sections were incubated overnight at 4°C in a humidified box with primary antibody Iba-1 (1:250). Then, the samples were incubated with secondary antibody: Alexa Fluor^®^ 488 AffiniPure Goat Anti-Rabbit IgG (Jackson; 1:200) for 2 h in the dark at room temperature. The following antibodies CD16/32 (1:250) and CD206 (1:250) were used overnight at 4°C. Next, the fluorescent secondary antibody Alexa Fluor^®^ 488 AffiniPure Donkey Anti-Goat IgG (1:200) was incubated for 2 h at room temperature in the dark at room temperature. In addition, DAPI was used to counterstain nuclei for 10 min in the dark. Finally, the sections were photographed using a laser confocal microscope (Leica TCS SP5, Germany). The merged yellow fluorescence intensity was detected by ImageJ software. And the relative quantification of each groups were based on fluorescence intensity.

### Cell Culture and Oxygen-Glucose Deprivation

The BV2 microglia (Concord Cell Resource Center, China) growth in 90% high glucose Dulbecco’s Modified Eagle Medium (DMEM) medium supplemented with 10% fetal calf serum (Gibco, United States), 1% penicillin-streptomycin (Gibco, United States), and cells were cultured at 37°C in humidified 5% CO2/95% air for 7–10 days prior to experimentation. The liquid was changed every other day.

BV2 microglia were exposed to OGD for establishing an *in vitro* model of cerebral ischemia. Before exposure, cultured cells were pretreated with Tanshinone IIA (10 μM) and then the cells seeded in glucose free DMEM medium (Gibco, United States) transferred into an anoxic device (Billups Rothenberg, United States), and flushed of a 95% N_2_ and 5% CO_2_ gas mixture for 4 h. Control group cells grow normally in the incubator.

### Cell Counting Kit-8 Assay

A Cell Counting Kit-8 (Beyotime, China) was used to assess cell survival. The experimental steps were strictly performed according to the manufacturer’s manual. Briefly, BV2 cells were plated at a density of 1.0 × 10^5^ cells/ml in 96-well cell culture plates. 10 μl of CCK-8 solution was added to each culture well of a 96-well plate and incubated for 1–4 h at 37°C. The absorbance at 450 nm was measured with a microplate reader (Multiskan MK3; Thermo Fisher Scientific, Inc.).

### Enzyme-Linked Immunosorbent Assay

The ischemic hemibrain tissue were placed in PBS and were homogenized, then supernatants were obtained by centrifugation at 12,000 r/min, 4°C for 15 min. The levels of interleukin-6 (IL-6), tumor necrosis factor-α (TNF-α), and interleukin-1 β (IL-1β) were detected by the corresponding ELISA kits (R&D, United States) according to the manufacturer’s instructions. Measurements were made using a microplate reader (Multiskan MK3; Thermo Fisher Scientific, United States).

### Superoxide dismutase and Malondialdehyde Measurement

The ischemic hemibrain tissue were placed in PBS and then supernatants were obtained by centrifugation at 12.000 r/min, 4°C for 15 min. The levels of SOD were detected by the SOD kit (Jiancheng Bioengineerinng, China). The levels of MDA were detected by the MDA kit (Jiancheng Bioengineerinng, China).

### Real-Time Polymerase Chain Reaction Assay

BV2 microglia cells were collected from each group. Total RNA was isolated as following: Add 500 μl of RNAiso Trizol (Takara, Japan) to 100 μl of chloroform (Sinopharm, China) for 5 min. Centrifuge for 15 min. Add equal volume of isopropanol to the supernatant and mix for 10 min. Centrifuge for 10 min, add 1 ml of 75% ethanol (Sinopharm, China), and centrifuge for 5 min. Add RNase-free H_2_O (Takara, Japan) to dissolve. All centrifugation was carried out at 12,000g at 4°C. After the RNA concentration was determined by ultraviolet absorption. Then, 1000 ng of total RNA was reverse transcribed in a 20-μl reaction at 37°C for 15 min followed by 85°C for 5 s using PrimeScript RT Master Mix (Takara, Japan). Real-time PCR was performed in a 20-μl reaction according to the manufacturer’s manual for SYBR Premix Ex Taq (Takara, Japan). The primer sequences were as follows:

GAPDH Forward: 5′-CTT​CAC​CAC​CAT​GGA​GAA​GGC-3′
Reverse: 5′-GGC​ATG​GAC​TGT​GGT​CAT​GAG-3′
CD11b Forward: 5′-CCA​AGA​CGA​TCT​CAG​CAT​CA-3′
Reverse: 5′-TTC​TGG​CTT​GCT​GAA​TCC​TT-3′
CD32 Forward: 5′-AAT​CCT​GCC​GTT​CCT​ACT​GAT​C-3′
Reverse: 5′-GTG​TCA​CCG​TGT​CTT​CCT​TGA​G-3′
iNOS Forward: 5′-GGC​AGC​CTG​TGA​GAC​CTT​TG-3′
Reverse: 5′-GCA​TTG​GAA​GTG​AAG​CGT​TTC-3′
Arg-1 Forward: 5′-GAA​CAC​GGC​AGT​GGC​TTT​AAC-3′
Reverse: 5′-TGC​TTA​GCT​CTG​TCT​GCT​TTG​C-3′
CD206 Forward: 5′-TTC​GGT​GGA​CTG​TGG​ACG​AGC​A-3′
Reverse: 5′-ATA​AGC​CAC​CTG​CCA​CTC​CGG​T-3′
IL-10 Forward: 5′-TAA​CTG​CAC​CCA​CTT​CCC​AG-3′
Reverse: 5′-AGG​CTT​GGC​AAC​CCA​AGT​AA-3′

The reaction was performed at 95°C for 30 s followed by 45 cycles of 95°C for 15 s and 55°C for 5 s, 72°C, 5 s on the Real-Time PCR Detection System (AB7500, United States). The relative mRNA expression were analyzed by the 2^−ΔΔCt^ method.

### Western Blot Analysis

The BV2 cells were collected, and the total cellular protein was extracted by standard extraction reagent (Thermo, United States) supplemented with protease inhibitors (Merck, Germany) and phosphatase inhibitor (Merck, Germany). Protein concentration was determined by bicinchoninic acid protein assay kit (Pierce, Thermo, United States). The extracted proteins were separated by SDS-PAGE and electrically transferred to nitrocellulose membranes (Millipore, United States). Then, the membranes were blocked with Tris-buffered saline with 5% non-fat milk for 1–2 h at room temperature. The following primary antibodies were used: iκB (1:1,000), p-iκB (1:1,000), NF-κB (p65) (1:1,000), p-NF-κB (p-p65) (1:1,000), GAPDH (1:1,000) from Abcam and Cell signaling technology. The membranes were shaken at 4°C overnight and incubated with fluorescent secondary antibody: Alexa Fluor^®^ 488 AffiniPure Goat Anti-Rabbit IgG (1:10,000) for 1 h at room temperature in dark. Protein bands were visualized using the Odyssey Imager with Odyssey 1.1 software (Li-Cor). Digital images were quantified using densitometry measurements by ImageJ software.

### Statistical Analysis

The statistical analysis was performed using SPSS software. Data were expressed as the mean ± standard deviation. Comparison among groups was analyzed by one-way analysis (One-way ANOVA) of variance. *p* < 0.05 was considered statistically significant.

## Results

### Tanshinone IIA Ameliorated Neurological Deficits and Reduced Infarct Volumes in Rats

**FIGURE 1:**
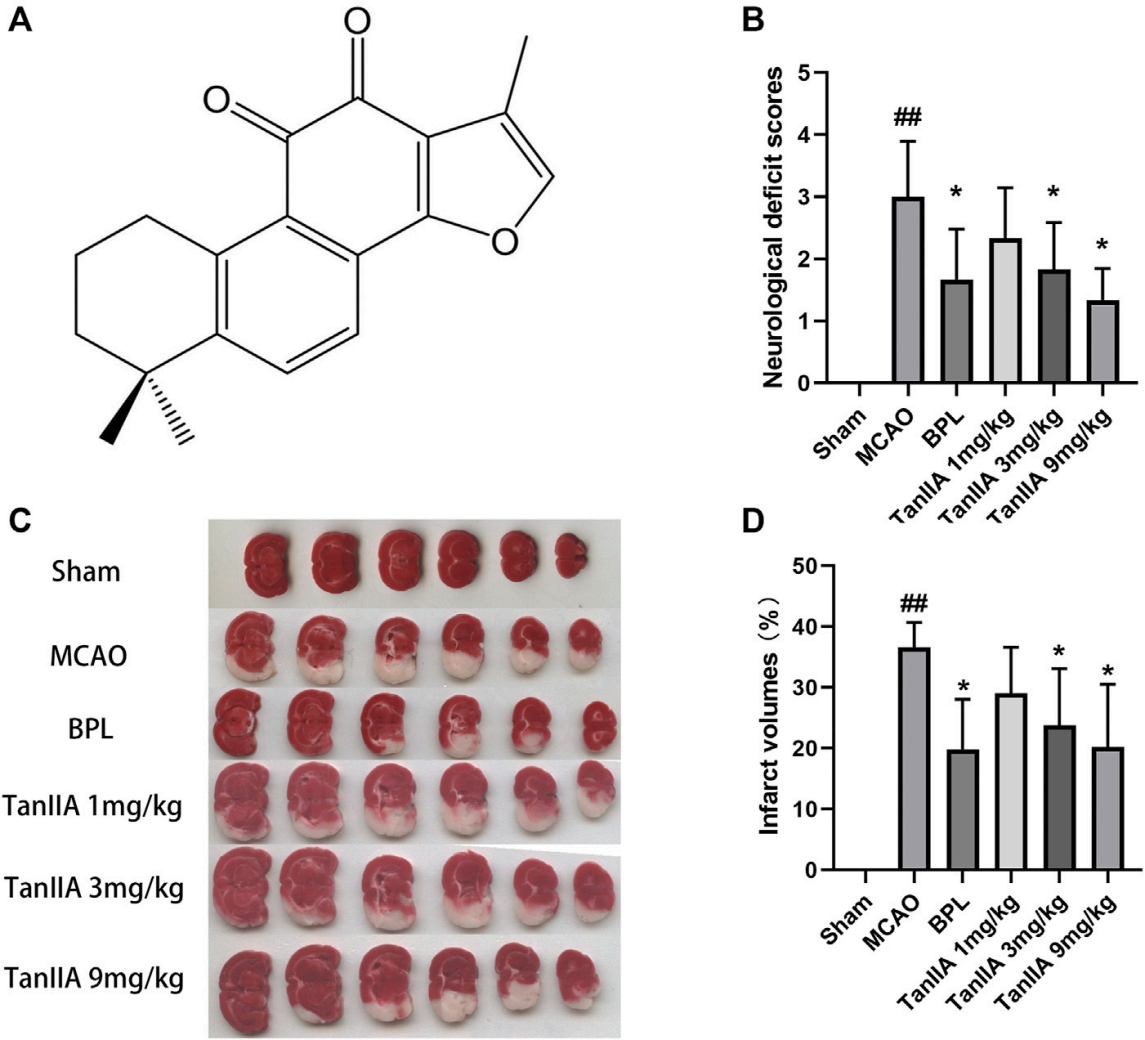
Tanshinone IIA treatment ameliorated neurological deficits and reduced infarct volumes in rats. Tanshinone IIA, BPL or control saline was injected into the tail vein at the indicated time after occlusion. After 24 h, the neurological deficit scores were measured, and the rats were euthanatized and the brain sections were stained with TTC for infarct volume measurement. **(A)** The molecular structure of Tanshinone IIA. **(B)** The neurological deficit scores of rats in each group. **(C)** TTC staining in each group. **(D)** Statistical results of cerebral infarct volumes in each group. ##*p* < 0.01, compared with the Sham group. *p < 0.05, compared with the MCAO group. Sham, sham operation group; MCAO, middle cerebral artery occlusion; BPL, butyphthalide (positive control); TanIIA, Tanshinone IIA, *n* = 6.

The molecular structure of Tanshinone IIA, 1,6,6-trimethyl-6,7,8,9-tetrahydrophenanthro[1,2-b] furan-10,11-dione, was shown in Figure 1A. BPL and Tanshinone IIA (3 mg/kg, 9 mg/kg) decreased the neurological deficit scores in rats (*p* < 0.05; Figure 1B). Additionally, the infarct volumes of rat brains were measured with TTC staining (Figure 1C).

Tanshinone IIA treatment at 1 mg/kg dose did not decrease the cerebral infarct volume compared to the MCAO group. However, when used at higher doses (3 and 9 mg/kg), it induced a significant neuroprotection after MCAO (*p* < 0.05; Figure 1D) (Two rats died in the MCAO group and one in the Tanshinone IIA (1 mg/kg) group after 24 h of reperfusion).

### Tanshinone IIA Alleviated the Inflammatory Response and Oxidative Damage

**FIGURE 2:**
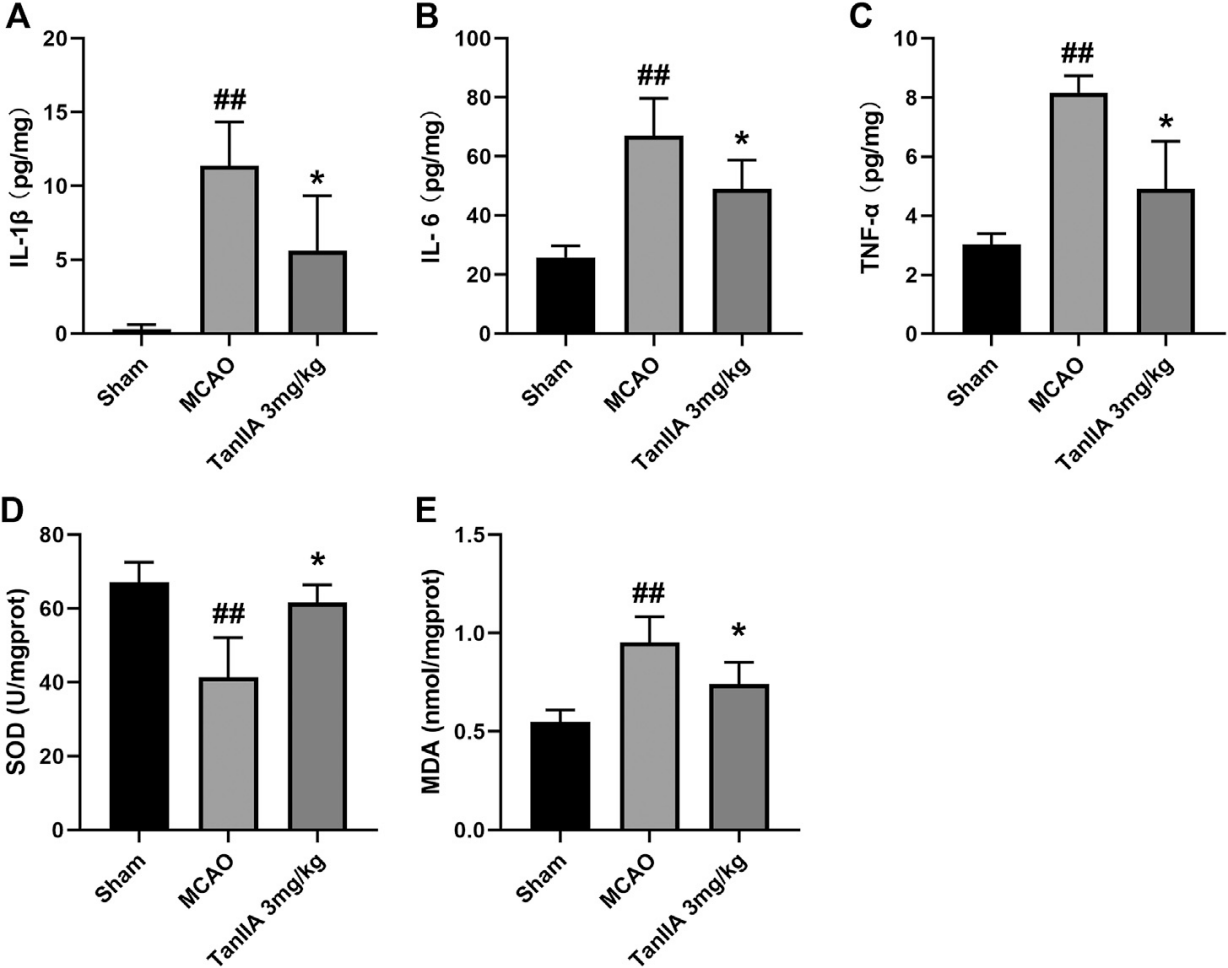
Tanshinone IIA inhibited inflammatory cytokines and Oxidative Damage in MCAO rats. Tanshinone IIA or control saline was injected into the tail vein at the indicated time after MCAO. After reperfusion, the rat brains were harvested. ELISA was used to detect levels of IL-1β **(A)**, IL-6 **(B)** and TNF-α **(C)**. SOD kit and MDA kit were used to detect the levels of SOD **(D)** and MDA **(E)**. ##*p* < 0.01, compared with the Sham group, *p < 0.05, compared with the MCAO group. Sham, sham operation group; MCAO, middle cerebral artery occlusion; TanIIA, Tanshinone IIA, *n* = 3.

After 24 h reperfusion, the expression levels of pro-inflammatory cytokines were detected by Enzyme-linked Immunosorbent Assay (ELISA). Compared with the sham group, MCAO treatment significantly upregulated levels of IL-1β (*p* < 0.01; Figure 2A), IL-6 (*p* < 0.01; Figure 2B), and TNF-α (*p* < 0.01; Tanshinone IIA 3 mg/kg treatment group vs. MCAO group; Figure 2C) in rat cerebral cortex. Tanshinone IIA (3 mg/kg) inhibited the MCAO-induced inflammatory cytokines and reversed the decrease of SOD (*p* < 0.05; Tanshinone IIA 3 mg/kg treatment group vs. MCAO group; Figure 2D) and the increase of MDA (*p* < 0.05; Tanshinone IIA 3 mg/kg treatment group vs. MCAO group; Figure 2E) in rats ischemic brain tissues. These results suggest that Tanshinone IIA can reduce the expression of oxidative damage and inflammatory factors.

### Pathological Changes and Apoptosis Levels in Rat Cortex

**FIGURE 3:**
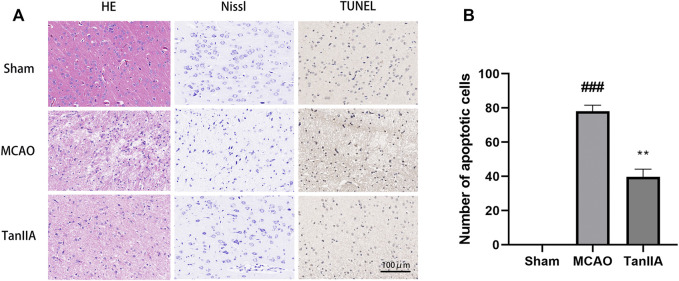
Effects of Tanshinone IIA on pathology and apoptosis after MCAO in rat brains. Tanshinone IIA or control saline was injected into the tail vein at the indicated time after MCAO. After reperfusion, the rat brains were harvested. **(A)** Pathological changes of rat cortex tissues were detected by HE staining and Nissl staining, apoptotic cells in rat cortex were detected by TUNEL staining (Scale bar: 100 μm). **(B)** The apoptotic cells of different groups were calculated and shown. ###*p* < 0.001, compared with the Sham group, **p < 0.01, compared with the MCAO group. Sham, sham operation group; MCAO, middle cerebral artery occlusion; TanIIA, Tanshinone IIA, *n* = 3.

Next, we examined the protective effects of Tanshinone IIA on cell injury in the rat brain tissues after MCAO. H&E staining was used to check the morphological changes. As showed in Figure 3A, cells of Sham group were neatly arranged, showing full cell morphology and distinct nuclei. In the MCAO group, most neurons in the cerebral cortex’s ischemic penumbra presented cell edema and nucleus solidification. Contrary to the MCAO group, cortical neuronal cell vacuolization was reduced, and cell morphology returned to normal in the Tanshinone IIA treatment group. The important components of neurons are the neuron nucleosomes, which are mainly composed of nucleic acids, ribose, and proteins, and are closely related to neuronal function. Nissl staining indicated that a large number of cells were shrunk down with an enlarged intercellular space and more dark color staining in the MCAO group relative to the Sham group. Meanwhile, these characteristic changes were all improved in the Tanshinone IIA treatment group. Furthermore, TUNEL staining was used to detect apoptosis in rat brains (Figure 3A). Apoptotic bodies stained brown were not observed in brain tissue cells of the Sham group, and a large number of apoptotic bodies were observed in the MCAO group. By counting the apoptotic cells in each group, we found that the number of apoptotic cells in the Tanshinone IIA treatment group was significantly reduced compared with the MCAO group (*p* < 0.01, Figure 3B). These results suggested that Tanshinone IIA relieves the damage caused by cerebral ischemia.

### Tanshinone IIA Reduced Hypoxia-Induced Neuronal Cell Death

**FIGURE 4:**
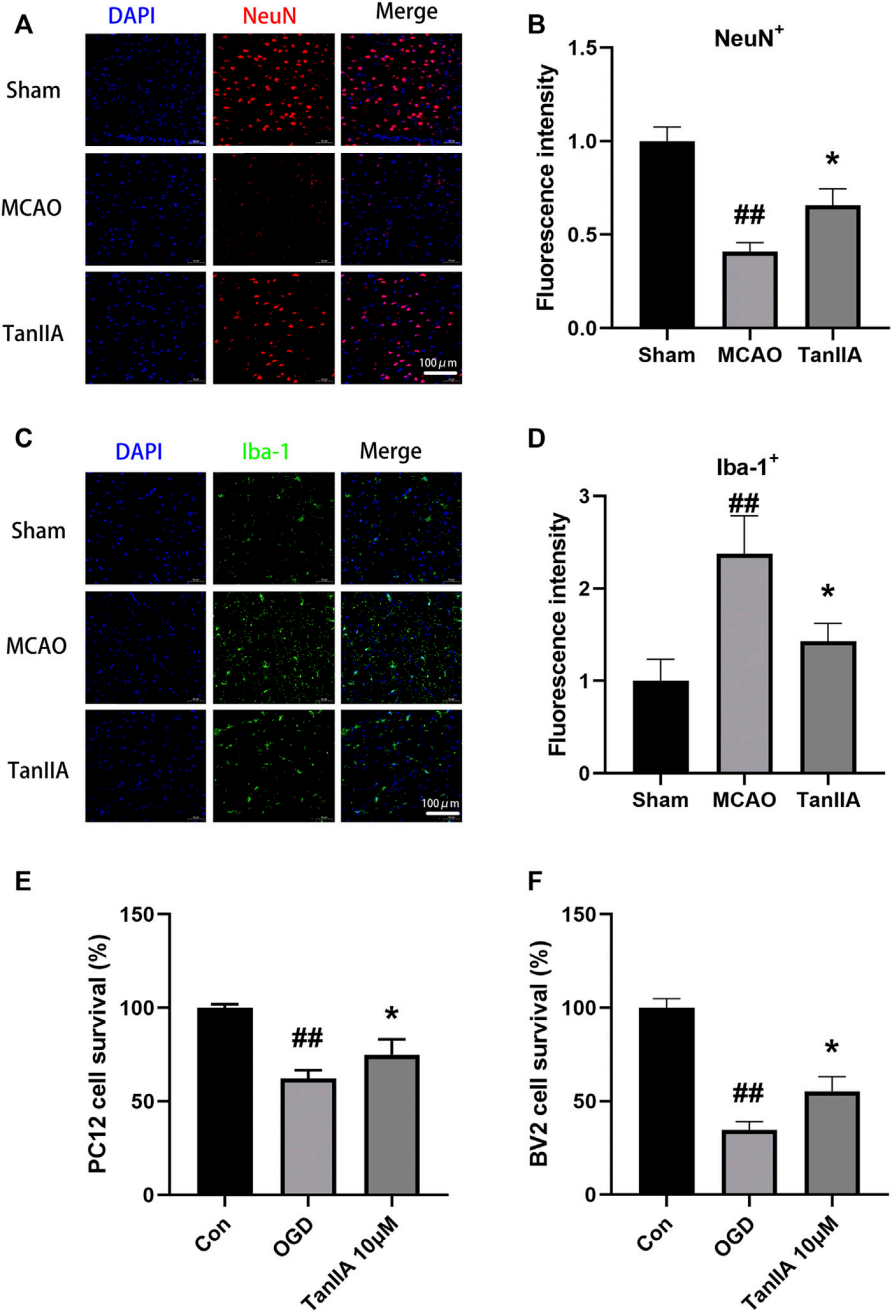
Effects of Tanshinone IIA on neuroprotection *in vivo* and *in vitro*. Immunofluorescence staining was used to detect the levels of NeuN-positive neuron **(A)** and Iba-1-positive microglia **(C)** in rat ischemic penumbra area. NeuN was shown as red, Iba-1 was shown as green and DAPI was shown as blue (Scale bar: 100 μm). **(B)** Fluorescence intensity of NeuN-positive neuron in rat ischemic penumbra area. **(D)** Fluorescence intensity of Iba-1-positive microglia in rat ischemic penumbra area. ##*p* < 0.01, compared with the Sham group, *p < 0.05, compared with the MCAO group. Sham, sham operation group; MCAO, middle cerebral artery occlusion; TanIIA, Tanshinone IIA, *n* = 3. OGD-induced BV2 cells were treated with Tanshinone IIA. CCK-8 assay was used to measure the cell survival of PC12 cells **(E)** and BV2 cells **(F)** after oxygen-glucose deprivation (OGD). ##*p* < 0.01, compared with the control group, *p < 0.05, compared with the OGD group. Con, control group; OGD, oxygen-glucose deprivation; TanIIA, Tanshinone IIA, *n* = 6.

The results of immunofluorescence showed that the fluorescence intensity of NeuN-positive neuron was decreased in the MCAO group. By calculating the fluorescence intensity of each group, we found that Tanshinone IIA significantly increased the fluorescence intensity of NeuN-positive neuron in rat cortex (*p* < 0.05; Tanshinone IIA 3 mg/kg treatment group vs. MCAO group; Figures 4A,B). Meanwhile, compared with the sham group, the fluorescence intensity of Iba-1-positive microglia was significantly increased in the MCAO group (*p* < 0.01). After Tanshinone IIA treatment, the fluorescence intensity of Iba-1-positive microglia reduced compared to the MCAO group (*p* < 0.05, Figures 4C,D).

Next, we investigated the protective effect of Tanshinone IIA on PC12 cells and BV2 cells exposed to hypoxia. We established an OGD model with a hypoxia time of 4 h. CCK-8 assay was used to detect the cell viability. Compared with the OGD group, Tanshinone IIA treatment group significantly increased the cell survival of PC12 cells and BV2 cells (*p* < 0.05; Tanshinone IIA 3 mg/kg treatment group vs. OGD group; Figures 4E,F). These results were indicated that Tanshinone IIA reduced hypoxia-induced neuronal cell death.

### Tanshinone IIA Regulated the Polarization of Microglia *in Rats*

**FIGURE 5:**
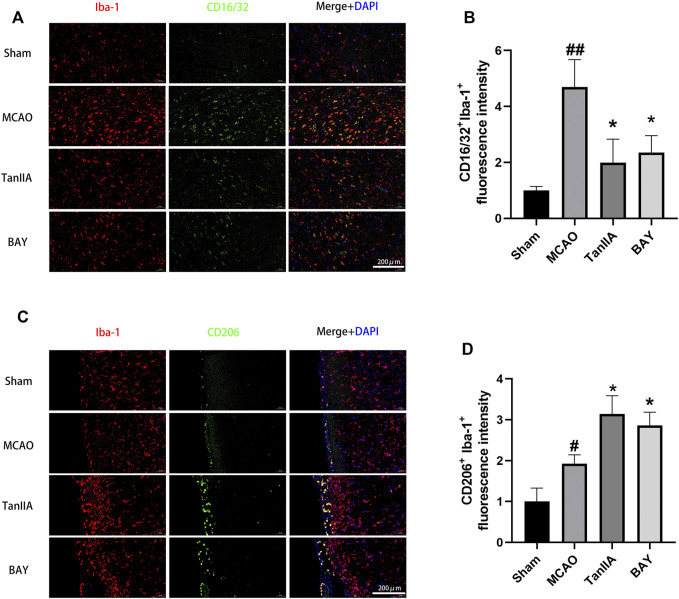
Tanshinone IIA regulated the polarization of microglia in rat cortex. Tanshinone IIA or control saline was injected into the tail vein at the indicated time after MCAO. After reperfusion, the rat brains were harvested. **(A)** Co-immunofluorescence staining of rat brain tissues for microglia marker Iba-1 (red) and M1 marker CD16/32 (green), **(B)** and the level of CD16/32^+^ Iba-1^+^ cells were analyzed and shown. **(C)** Co-immunofluorescence staining of rat brain tissues for microglia marker Iba-1 (red) and M2 marker CD206 (green), **(D)** the level of CD206^+^ Iba-1^+^ cells were analyzed and shown. ##*p* < 0.01, compared with the Sham group, *p < 0.05, compared with the MCAO group. Sham, sham operation group; MCAO, middle cerebral artery occlusion; TanIIA, Tanshinone IIA, BAY, BAY-11-7082, a NF-κB inhibitor, *n* = 3. (Scale bar: 200 μm).

After cerebral ischemia, microglia were activated and polarized into pro-inflammatory M1 and anti-inflammatory M2. Co-immunofluorescence staining was performed to observe the polarization of M1 and M2 microglia in rat brains (Figures 5A,C). The result of fluorescence intensity assay showed that Tanshinone IIA and BAY-11-7082 (a NF-κB inhibitor) treatments down-regulated CD16/32^+^Iba-1^+^ microglia cells activated by MCAO (*p* < 0.05; Tanshinone IIA 3 mg/kg treatment group vs. MCAO group; Figure 5B). Meanwhile, Tanshinone IIA and BAY-11-7082 (a NF-κB inhibitor) treatments upregulated CD206^+^Iba-1^+^ microglia cells compared with the MCAO group (*p* < 0.05; Tanshinone IIA 3 mg/kg or BAY-11-7082 3 mg/kg treatment group vs. MCAO group; Figure 5D).

### The mRNA Expression Levels of BV2 Cells After Oxygen-Glucose Deprivation

**FIGURE 6:**
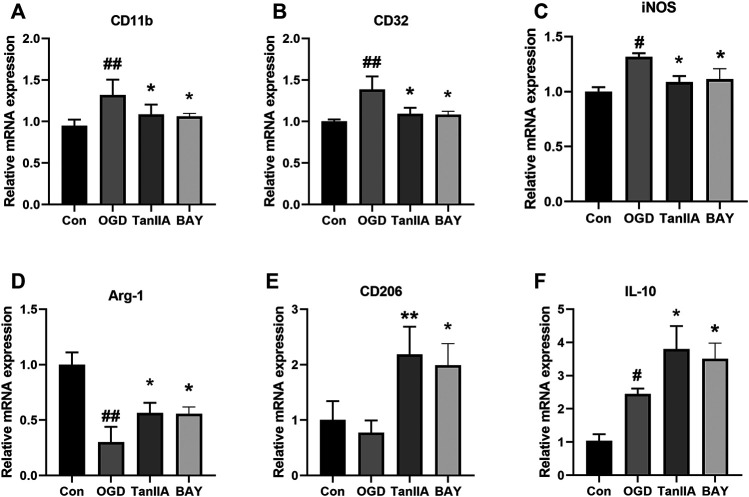
Effect of Tanshinone IIA on polarization-related mRNA expressions after OGD in BV2 cells. OGD-induced BV2 cells were treated with Tanshinone IIA. Quantitative RT-PCR was used to assess the mRNA expression levels of M1 microglia markers: CD11b **(A)**, CD32 **(B)**, iNOS **(C)**, and M2 microglia markers: Arg-1 **(D)**, CD206 **(E)**, IL-10 **(F)**. ##*p* < 0.01, compared with the Con group, *p < 0.05, compared with the OGD group. Con, control group; OGD, oxygen-glucose deprivation; TanIIA, Tanshinone IIA. BAY, BAY-11-7082, a NF-κB inhibitor, *n* = 3.

To further investigate the effect of Tanshinone IIA on microglia, we used quantitative RT-PCR to detect the mRNA expression levels of M1 and M2 markers in BV2 cells. Tanshinone IIA and BAY-11-7082 (a NF-κB inhibitor) downregulated the mRNA expressions of M1 markers in OGD-induced BV2 cells: CD11b, CD32, iNOS (*p* < 0.05; Tanshinone IIA 10 μM or BAY-11-7082 5 μM treatment group vs. OGD group; Figures 6A–C), and upregulated the mRNA expression levels of M2 markers: Arg-1, CD206 and IL-10 (*p* < 0.05; Tanshinone IIA 10 μM or BAY-11-7082 5 μM treatment group vs. OGD group; Figures 6D–F). The above data were shown that Tanshinone IIA and BAY-11-7082 could inhibit M1 type and promote M2 type of microglia's polarization.

### Tanshinone IIA Regulated the Polarization of Microglia by NF-κB Signaling Pathway

**FIGURE 7:**
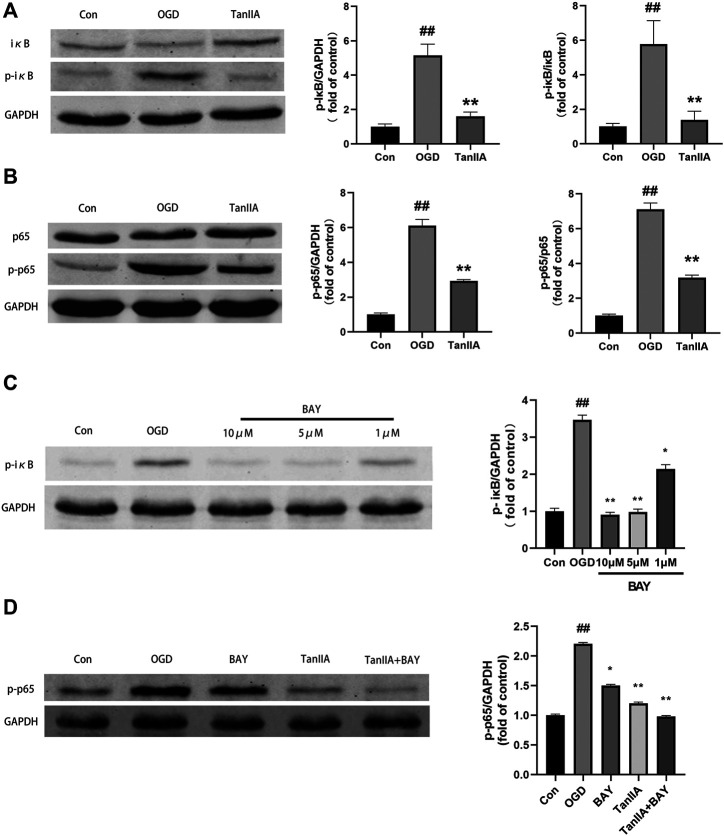
Tanshinone IIA regulated NF-κB signaling pathway in BV2 cells after oxygen-glucose deprivation (OGD). **(A)** The protein level of p-IκB in BV2 cells treated with Tanshinone IIA. **(B)** The protein level of p-p65 in BV2 cells treated with Tanshinone IIA. **(C)** The protein level of p-IκB in BV2 cells treated with BAY-11-7082. **(D)** The protein level of p-p65 in BV2 cells treated with Tanshinone IIA and BAY-11–7082. ##*p* < 0.01, compared with the control group. *p < 0.05, **p < 0.01, compared with the OGD group. Con, control group; OGD, oxygen-glucose deprivation; TanIIA, Tanshinone IIA; BAY, BAY-11-7082, a NF-κB inhibitor, *n* = 3.

**FIGURE 8:**
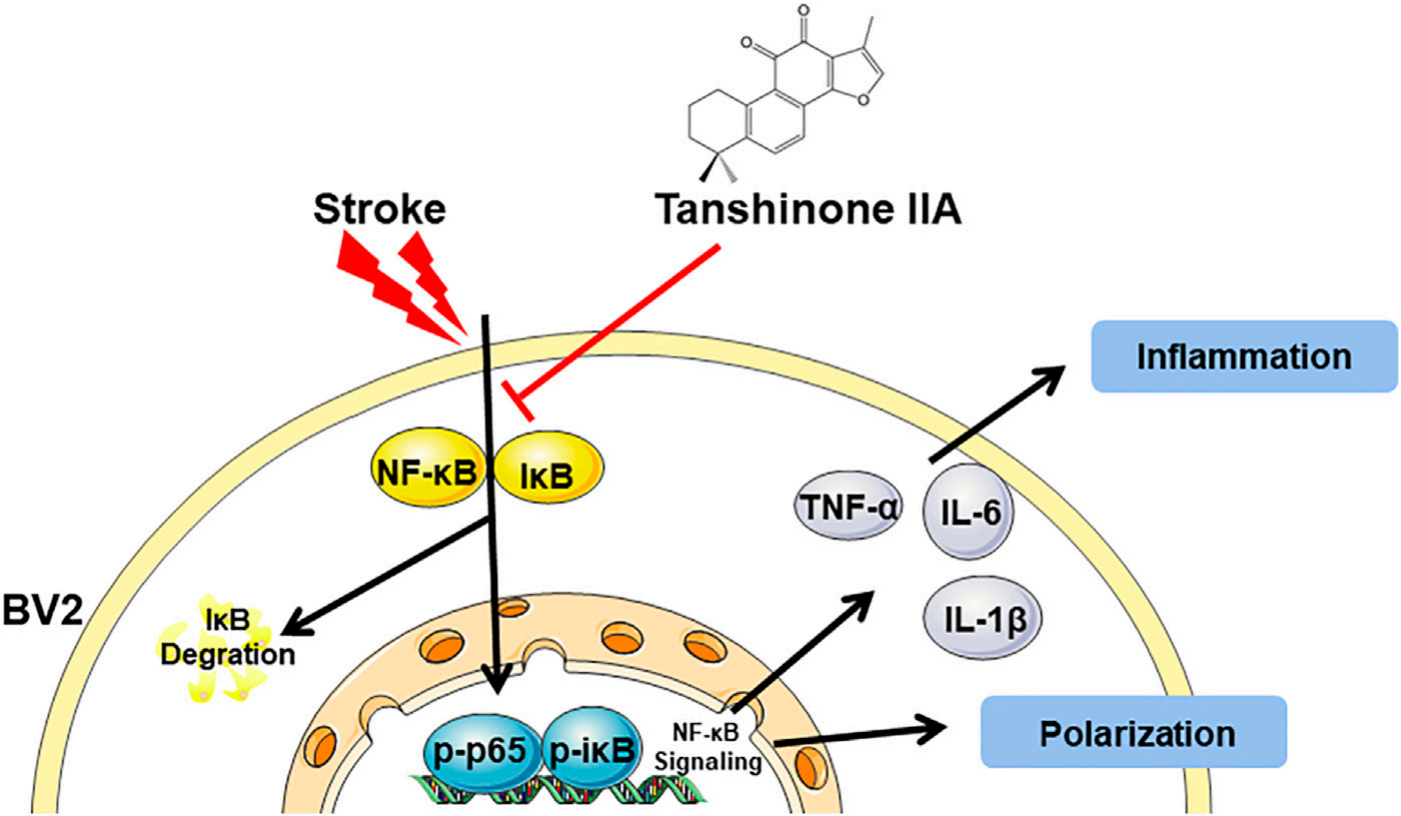
A graphical representation of the neuroprotective mechanisms of Tanshinone IIA against the stroke-induced neuroinflammation by NF-κB signaling pathway.

Previous studies have shown that the NF-κB signaling pathway has an important role in inflammatory signaling (Bruno et al., 2018). Western blot was used to detect the protein expression of NF-κB signaling pathway in BV2 cells after oxygen-glucose deprivation (OGD). Tanshinone IIA down-regulated the expression levels of p-iκB and p-p65 proteins in BV2 cells (*p* < 0.01; Tanshinone IIA 10 μM treatment group vs. OGD group; Figures 7A,B). In addition, BAY-11-7082 (a NF-κB inhibitor) also downregulated the expression of *p*-iκB and p-p65 in BV2 cells, and strengthened the inhibiton of p-p65 by Tanshinone IIA (*p* < 0.05; BAY-11-7082 5 μM treatment group vs. OGD group; Figures 7C,D). These results indicated that Tanshinone IIA may inhibit the polarization of microglia *via* NF-κB signaling pathway (Figure 8).

## Discussion

Stroke is a major cause of death and disability worldwide, and traditional Chinese medicine has high application value and therapeutic potential in stroke (Liu et al., 2018). In the previous research, Chen et al. (2012a) reported that intraperitoneal injection of Tanshinone IIA (25 mg/kg) can significantly reduce the brain infarct volume of cerebral ischemia in rats. And a recent study showed that Tanshinone IIA (20 and 40 mg/kg) reduced brain water content and normalized neurological deficit score after cerebral ischemia-reperfusion injury in rats (Wang et al., 2020). In this study, we found that tail vein injection of Tanshinone IIA (3 and 9 mg/kg) significantly reduced the brain infarct volume and normalized neurological deficit score after cerebral ischemia-reperfusion injury in rats. Furthermore, H&E staining and Nissl staining were used to detect the pathological changes in rat brain tissues. The results showed that Tanshinone IIA treatment significantly improved the distribution and morphological structure of neurons. TUNEL staining showed that Tanshinone IIA treatment can reduce apoptotic cells in rat brain after MCAO. These results indicated that Tanshinone IIA can moderate the cerebral ischemia damage in rats.

The inflammatory response has an important role in cerebral ischemia-reperfusion injury, and most of the damage is caused by a secondary inflammatory cascade. Overexpression of inflammatory factors, such as TNF-α, IL-6, IL-1β, promotes the inflammatory response’s progression (Tobin et al., 2014; Ai et al., 2017; Wei et al., 2017). In acute cerebral ischemic injury, the excessive formation of oxygen free radicals leads to excessive oxidative stress, which, in turn, leads to cells and tissue damage (Naderi et al., 2017). SOD, as a major antioxidant enzyme, represents the strength of the antioxidant system. MDA is a product of lipid peroxidation in the cell membrane, which indicates the degree of damage caused by oxidative stress (Sun et al., 2016). Our results showed that Tanshinone IIA could inhibit the expression of pro-inflammatory factors TNF-α, IL-6, and IL-1β and regulate the levels of SOD and MDA in the brain tissue of rats with cerebral ischemia.

Microglia, representing the brain’s major innate immune cells, play an important role in neuroinflammation (Pape et al., 2019). Affected by the environment, microglia have various phenotypes, which participate in innate immunity and the central nervous system (Orihuela et al., 2016). Under normal conditions, they clear cellular debris and toxic cells by phagocytosis. Once these cells are activated, they transmit and translate peripheral-initiated inflammatory signaling factors (Yenari et al., 2010). Previous studies have found that resveratrol promotes neuronal protection by limiting inflammatory cytokines production, thereby reducing induced microglial activation (Zhao et al., 2018). In the activation of BV2 and primary glial cells, some scholars have found that ingredients in Cudrania tricuspidata can inhibit the production of pro-inflammatory factors in cells against acute neuroinflammation (Kim et al., 2017). After activation, microglia can be divided into classically activated microglia (M1) and selectively activated microglia (M2) (Orihuela et al., 2016). M1 type can produce pro-inflammatory factors and mediators (IL-1β, IL-6, TNF-α, CCL2, ROS, NO, glutamate, etc.), resulting in peripheral inflammatory cell infiltration. They also initiate an inflammatory response, causing apoptosis and secondary damage (Devanney et al., 2020). In contrast, activated M2 cells produce an anti-inflammatory cytokine IL-10 that inhibits neuronal inflammation, expressing tissue remodeling receptors (Arg-1, CD36, CD163, CD206, etc.), transforming growth factor β (TGF-β), brain-derived neurotrophic factor (BDNF) (Boche et al., 2013; Hu et al., 2015), and help restore the homeostasis of the central nervous system (Gogoleva et al., 2019). Therefore, promoting microglia conversion to M2 type is considered a therapeutic strategy for cerebral ischemic injury (Lu et al., 2020). In this study, we found that Tanshinone IIA reduced ischemia-induced neuronal cell death by increasing neuron and microglia cell numbers *in vivo*. Then we established an OGD model of PC12 and BV2 cells to mimic the cerebral ischemic environment in rats. Tanshinone IIA increased the survival of PC12 and BV2 cells after OGD. In a recent study, Tanshinone IIA can shift microglia phenotype from M1 to M2 in A. cantonensis-infected BALB/c mice (Feng et al., 2019). Tanshinone IIA treatment downregulated M1 macrophage markers and upregulated M2 macrophage markers in BV-2 cells under LPS stimulation (Chen et al., 2020). Next in our experiments, co-immunofluorescence staining was used to detect the polarization of microglia in rat brain. Interestingly, we found that Tanshinone IIA could inhibit the M1 type of microglia’s polarization and promote the polarization of the M2 type in rat cortex. In addition, we also found that Tanshinone IIA could significantly downregulated the mRNA expressions of M1 markers in OGD-induced BV2 cells: CD11b, CD32, iNOS and upregulated the mRNA expression levels of M2 markers: Arg-1, CD206 and IL-10. These results suggest that Tanshinone IIA may play a neuroprotective role by regulating microglia polarization. However, its mechanism was unknown.

NF-κB factors have been shown to regulate cell survival and proliferation, inflammation, and immune response processes (Pires et al., 2018). Its protein products p65/p50 and Rel/p52 are relatively stable dimers (Huang et al., 1997; Cai et al., 2020). The function of NF-κB members may depend on specific cellular stimuli (Freire and Conneely, 2018). Pattern recognition receptors, cytokine or antigen receptor-mediated signaling cascades eventually culminate in the activation of the IκB kinase (IKK) complex, followed by inhibition of intracellular NF-κB chaperone IκBα and related protein phosphorylation. This phosphorylation results in ubiquitination and degradation of K48-linked IκBα, thus allowing NF-κB dimers to translocate into the nucleus and initiate specific gene transcription, increase nuclear NF-κB, initiate inflammatory responses and downstream pathways (Liu et al., 2017; Zhang et al., 2018). The relationship between NF-κB and stroke was studied by detecting the expression of RelA and p50 in brain tissue in six patients after death (Harari and Liao, 2010). During cerebral ischemia, NF-κB is activated (Taetzsch et al., 2015), and M1 type microglia is polarized (Wang et al., 2017). Dong et al. (2009) reported that Tanshinone IIA inhibited the translocation of NF-κB in the nucleus on H_2_O_2_ induced astrocytes. And Wang et al. (2014) reported that Tanshinone IIA reduced the expression of NF-κB in rat brain after I/R. In this study, we examined proteins associated with the NF-κB pathway, finding that Tanshinone IIA inhibited expression levels of NF-κB signaling pathway-related proteins in BV2 cells. And further studies showed that BAY-11-7082 (a NF-κB inhibitor) could also inhibit M1 type and promote M2 type of microglia's polarization *in vivo* and *in vitro*. These results suggested that Tanshinone IIA may inhibit the inflammatory response by inhibiting M1 microglia polarization and promoting the polarization of the M2 type through the NF-κB pathway.

In conclusion, in this study, we again demonstrated that Tanshinone IIA is effective for the treatment of cerebral I/R injury in rats. We also found that Tanshinone IIA could modulate microglia polarization. Above all, the research suggested that Tanshinone IIA might regulate microglia polarization from M1 to M2 through NF-κB signaling pathway and thus have anti-neuroinflammatory effects.

## Data Availability

The original contributions presented in the study are included in the article/Supplementary Material, further inquiries can be directed to the corresponding authors.
